# The value of sperm DNA fragmentation testing in clinical practice: routine or case-directed?

**DOI:** 10.1093/hropen/hoag087

**Published:** 2026-10-09

**Authors:** Ahmad Majzoub, Peter Humaidan

**Affiliations:** Department of Urology, Hamad Medical Corporation, Doha, Qatar; Department of Clinical Urology, Weill Cornell Medicine-Qatar, Doha, Qatar; Department of Clinical Medicine, Aarhus University, Aarhus, Denmark; University Clinic for Fertility, Skive Regional Hospital, Skive, Denmark

Dear Editor,

We read with interest the recently published Opinion paper by Vitthala *et al*. in *Human Reproduction Open* that questions the routine use of sperm DNA fragmentation (SDF) testing in fertility clinics (Vitthala *et al*., 2026a). The authors provide a structured evaluation of SDF through screening, diagnostic, and prognostic test-performance frameworks and appropriately highlight limitations, including assay heterogeneity, inconsistent thresholds, and the paucity of high-quality interventional evidence.

However, we believe that several aspects of the article’s interpretation deserve further discussion, particularly as the authors appear to adopt an overly restrictive interpretation of clinical utility.

First, the evaluation of SDF as a population-level screening test is conceptually problematic. SDF testing until now has not generally been advocated as a screening tool for male infertility. Rather, it has most commonly been proposed as an adjunctive sperm function test in selected infertile couples or defined clinical scenarios (Agarwal *et al*., 2017; Esteves *et al*., 2021; Schlegel *et al*., 2021). Therefore, showing that SDF does not satisfy criteria for population screening does not directly address the more relevant question of whether SDF testing has clinical value when used selectively and interpreted within an appropriate reproductive context.

Furthermore, the Wilson–Jungner framework is primarily condition-centered (Wilson and Jungner, 1968). It asks whether a disease or condition is suitable for screening, considering factors such as disease importance, natural history, latent stage, accepted treatment, and benefit from early detection. SDF, however, is not a disease entity; it is a biomarker of sperm genomic integrity. If a screening framework is invoked, the initial question should be whether male infertility, or a clearly defined male infertility phenotype, is suitable for screening. This is inherently complex because infertility is a couple-dependent reproductive outcome rather than a simple male-only disease state. Female age, ovarian reserve, tubal and uterine factors, embryo quality, intercourse frequency, life style, and ART modality all influence reproductive outcomes (Practice Committee of the American Society for Reproductive Medicine, 2021). Consequently, screening concepts cannot be applied to ‘male infertility’ or SDF testing in a simplistic manner.

It is also worth noting that the criteria presented in Tables 1, 2, and 4 of Vitthala *et al*. (2026a) appear to be author-derived applications of established frameworks rather than outputs of validated scoring systems. Wilson–Jungner (Wilson and Jungner, 1968), STARD (Bossuyt *et al*., 2003), Fryback and Thornbury (Fryback and Thornbury, 1991), and prognostic model evaluation frameworks provide important principles for evaluating screening, diagnostic accuracy, clinical utility, discrimination, calibration, and net benefit. However, they do not provide the specific checkmark/cross scoring system used in the Vitthala *et al*. (2026a) Opinion article. Several judgments in the tables, including actionability, added value, treatment availability, early benefit, and clinical utility, are context-dependent and open to interpretation. Presenting these assessments in a binary or semi-binary format may therefore give an impression of objectivity that is not fully supported by the underlying material.

The data reported in Table 3 in Vitthala *et al*. (2026a) also require clarification. In support of their argument regarding the diagnostic and prognostic accuracy of SDF testing, the authors state that the values in Table 3 were derived from Vitthala *et al*. (2026b). However, the cited meta-analysis (Vitthala *et al*. 2026b) reports results across natural conception, IUI, IVF, ICSI, and assay-specific subgroups, whereas Table 3 in Vitthala *et al*. (2026a) collapses the data into broad assay-level estimates for pregnancy and miscarriage. Moreover, the original meta-analysis appears to define true positives as men with high SDF who experienced no pregnancy or miscarriage (Vitthala *et al*., 2026b). Therefore, the sensitivity and specificity values for the ‘pregnancy’ outcome may actually reflect prediction of failure to achieve pregnancy rather than prediction of pregnancy itself. If these values were recalculated, pooled across settings, or extracted from supplementary model outputs, this should have been explicitly stated. This is particularly important because positive predictive values (PPV) and negative predictive values (NPV) are prevalence-dependent and may not be generalizable across heterogeneous natural conception and ART populations.

Taken together, these concerns suggest that Vitthala *et al*. (2026a) may conflate the absence of scientific evidence for universal implementation until now with evidence against selective clinical application. Although current data are largely observational, consistent associations between elevated SDF and adverse reproductive outcomes, particularly recurrent pregnancy loss, unexplained infertility, and repeated ART failure, suggest that SDF testing provides clinically relevant information. Moreover, the growing body of evidence regarding oxidative stress, sperm genomic integrity, and testicular sperm use in specific scenarios deserves a more balanced discussion.

Finally, the authors’ conclusion in Vitthala *et al*. (2026a) that SDF testing offers only limited incremental value may underestimate its role in patient counselling, risk stratification, and identifying potentially modifiable male factors. The key issue is whether SDF can provide meaningful adjunctive information when ordered for appropriate indications and interpreted by clinicians with expertise in male reproductive medicine.

Rather than framing the debate as routine versus non-routine use, future research should focus on defining the patient populations most likely to benefit from SDF-guided management and establishing whether such strategies improve clinically meaningful outcomes, particularly live birth and miscarriage reduction.
